# Effects of exosomes from different origins on osteoclasts

**DOI:** 10.1186/s13018-025-06439-y

**Published:** 2025-12-22

**Authors:** Meng Yuan, Tianyu Zhu, Chuan Wang, Qun Xia, Yue Wang

**Affiliations:** 1 School of Integrative Medicine, Tianjin University of Traditional Chinese Medicine, Tianjin, 301617 China; 2 Faculty of Medical Technology, Tianjin University of Traditional Chinese Medicine, Tianjin, 301617 China; 3 NHC Key Lab of Hormones and Development and Tianjin Key Lab of Metabolic Diseases, Tianjin Medical University Chu Hsien-I Memorial Hospital & Institute of Endocrinology, Tianjin, 300134 China; 4 Department of Orthopaedics, Tianjin First Central Hospital, Tianjin, China

**Keywords:** Osteoclasts, Exosomes, Stem cells, Tumor cells

## Abstract

Abnormal osteoclast function is closely related to the development of osteolytic diseases such as osteoporosis and tumor bone metastasis, which seriously affects patients’ quality of life. The cellular microenvironment is complex and variable, and exosomes, as an important pathway mediating intercellular communication, can be secreted by a variety of cells in the human body, which carry “cargo” that can affect the biological function of recipient cells. Osteoclasts receive a variety of cellular exosomes to regulate and influence the body’s bone homeostasis. In this narrative review, we summarize and discuss the effects of exosomes from different sources, such as stem cells, tumor cells, osteoblasts, vascular cells, hematopoietic cells and immune cells, on the function of osteoclasts based on a literature search of PubMed and Web of Science up to June 2025. These findings provide a new way of thinking for the targeted treatment of osteolytic diseases, and exosomes are expected to be potential biological agents for the treatment of related diseases, but there are still problems such as low targeting and unknown mechanisms.

## Introduction

Bone homeostasis is meticulously regulated through a delicate balance between osteoblast-mediated bone formation and osteoclast-mediated bone resorption [1]. The disruption of this equilibrium, often excessive osteoclast activity, is a hallmark of various osteolytic diseases, including osteoporosis and tumor bone metastases, which severely impair patients’ quality of life and pose a significant socioeconomic burden [2, 3]. While current pharmacological interventions for these conditions exist, they are often hampered by limited efficacy, adverse effects, and poor long-term outcomes [4]. This clinical challenge has spurred the exploration of novel therapeutic strategies, among which stem cell therapy initially emerged as a promising option. However, practical hurdles related to tumorigenicity, immunogenicity, and delivery have limited its clinical translation [5–8]. Consequently, attention has shifted towards the paracrine mechanisms of stem cells, particularly their secretion of extracellular vesicles (EVs) [9]. EVs, including exosomes (30–150 nm in diameter), microvesicles (100–1000 nm), and apoptotic bodies (1–5 μm), are nano-sized, membrane-bound particles that facilitate intercellular communication by shuttling bioactive molecules (e.g., proteins, miRNAs, lncRNAs) between cells [10, 11]. Crucially, the biological effect of EVs is intrinsically linked to their cellular origin. For instance, EVs derived from osteoblasts or bone marrow mesenchymal stem cells (BMSCs) typically promote anabolic bone formation, whereas those secreted by osteoclasts, immune cells, or tumor cells often exacerbate catabolic bone resorption [12]. This origin-dependent functionality positions exosomes as critical mediators in both the maintenance and disruption of bone homeostasis. In recent years, the role of exosomes in osteolytic diseases has garnered immense research interest. It is now evident that osteoclasts are not only active resorbers but also sophisticated recipients of exosomal signals from a vast network of cells within the bone microenvironment. However, a comprehensive synthesis of how exosomes from these diverse cellular sources collectively orchestrate and influence osteoclast function is lacking.

Therefore, the primary aim of this narrative review is to systematically consolidate and critically evaluate the current understanding of how exosomes derived from various origins—including stem cells (e.g., BMSCs, ADSCs), tumor cells, osteoblasts, vascular cells, hematopoietic cells, and immune cells—modulate osteoclast differentiation and activity. By elucidating the underlying mechanisms and discussing the associated therapeutic potential, this review seeks to provide a valuable framework for future research and the development of exosome-based diagnostics and therapeutics for osteolytic diseases.

### Search strategy

This narrative review is based on a comprehensive literature search conducted in the PubMed and Web of Science electronic databases for articles published from inception to June 2025. The search strategy utilized a combination of the following Medical Subject Headings (MeSH) terms and keywords: “exosome”, “extracellular vesicle”, “osteoclast”, “bone resorption”, “osteolysis”, coupled with specific cell types (e.g., “mesenchymal stem cell”, “cancer”, “osteoblast”, “macrophage”). The inclusion criteria were: (1) original research articles published in English; (2) studies that directly investigated the effect of exosomes or extracellular vesicles on osteoclast formation or function. Conference abstracts and studies not focusing on osteoclasts were excluded. The reference lists of retrieved articles were also manually screened to identify additional relevant publications.

## Effects of stem cell-derived exosomes on osteoclasts

**Fig. 1:**
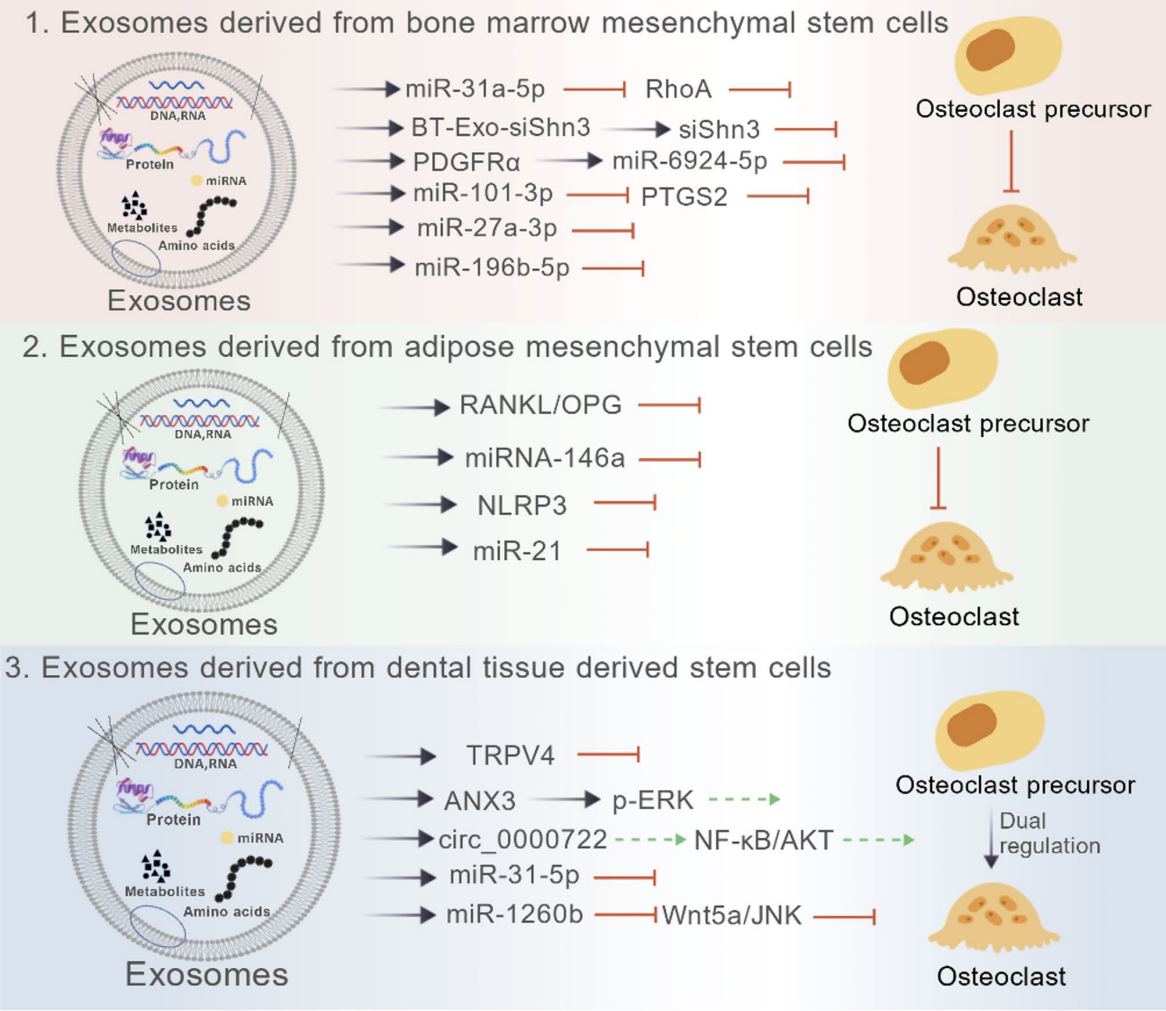
Effects of stem cell-derived exosomes on osteoclasts. (Created with BioGDP.com[49]).All molecules shown at the origin of the arrows (e.g., miR-31a-5p, BT-Exo-siShn3) represent the specific cargo encapsulated within the exosomes. The arrowheads indicate the downstream molecules or pathways that are targeted and regulated by this exosomal cargo in the recipient osteoclast precursors. (Black solid arrows: Targeted delivery relationships; Red T-bar arrows: Inhibition of osteoclast differentiation; Green dashed arrows: Promotion of osteoclast differentiation. Notably, exosomes derived from dental-tissue-derived stem cells exert dual regulation on osteoclasts, involving both promoting and inhibiting effects.)

Stem cells, particularly mesenchymal stem cells (MSCs), are a pivotal source of exosomes that play a regulatory role in bone remodeling. MSCs possess the capacity for self-renewal and multi-lineage differentiation, giving rise to osteoblasts, chondrocytes, and adipocytes, which underpins their significant role in maintaining bone homeostasis. The following sections detail the effects of exosomes derived from various types of MSCs on osteoclasts, as summarized in Fig. 1.

### Bone marrow mesenchymal stem cells

Stem cells have the ability of self-renewal and multi-lineage differentiation, and under certain conditions they can differentiate into cells with different functions [13]. Among them, mesenchymal stem cells are a type of adult stem cells with abundant resources. They have been widely applied in scientific research and play a significant role in maintaining bone homeostasis, as they are capable of differentiating into cells such as osteoblasts, chondrocytes, and adipocytes [14]. However, the clinical translation of MSCs themselves (including those derived from bone marrow, adipose tissue, and dental tissues) faces challenges, including potential risks such as phenotypic instability, immune-mediated rejection, and limited control over differentiation post-transplantation, which limit their clinical application [7, 15]. Fortunately, scientists have found that mesenchymal stem cells can also play their role in the treatment of diseases by secreting EVs, and at the same time, in contrast, extracellular vesicles (EVs), particularly exosomes derived from MSCs, have emerged as a promising cell-free therapeutic alternative. EVs are increasingly investigated due to their favorable properties, such as lower immunogenicity, a reduced risk of tumor formation compared to whole cells, high stability, and an innate capacity for tissue targeting. These attributes may potentially overcome several critical hurdles associated with conventional cell-based therapies [16]. Exosomes belong to a type of EVs, for instance, exosomes from aged bone marrow MSCs (BMSCs) were found to exhibit elevated levels of miR-31a-5p, and inhibition of miR-31a-5p in exosomes increased the transcription level of RhoA and reduced the production of osteoclasts, as demonstrated in a study where local injection of a miR-31a-5p antagonist reduced osteoclast differentiation in aged rats, suggesting that aging modulates exosomal miRNA profiles to promote osteoclastogenesis.. Inhibition of this miRNA increased RhoA transcription and suppressed osteoclast formation [9]. This result suggests that aging alters the expression levels of miRNAs in bone marrow mesenchymal stem cell exosomes and promotes osteoclast differentiation. Mesenchymal stem cell-derived exosomes are effective and feasible natural nano-drug delivery vehicles [17]. Beyond their natural cargo, MSC-derived exosomes can be engineered for enhanced therapeutic efficacy. For example, a bone-targeting exosome platform (BT-Exo-siShn3) was developed to deliver Shn3-specific siRNA, significantly inhibiting osteoclast multinucleation and exhibiting potent anti-osteoporotic effects both in vitro and in vivo [18, 19]. Similarly, exosomes from scleraxis-overexpressing PDGFRα( +) BMSCs inhibited osteoclast formation via delivery of miR-6924-5p, which targets OCSTAMP and CXCL12, thereby reducing osteolysis and promoting tendon-bone healing[20]. Mechanical stimulation is a regulatory factor in many physiological and pathological processes, and studies have found that mechanical stimulation can affect exosome secretion and the cargo carried by exosomes [21]. Prolonged bone weightlessness can cause severe disuse osteoporosis, which is common in patients who are bedridden for long periods of time or in astronauts in space. Xiao F et al*.* found that cyclic mechanical stretch-stimulated bone marrow mesenchymal stem cell-derived exosomes inhibited osteoclast production by inhibiting the activation of the NF-κB signalling pathway and alleviated mechanical weightlessness-induced bone loss in a hindlimb unloading mouse model [22]. In 2022, Wu et al. found that Morinda polysaccharide can upregulate the expression of miR-101-3p in bone marrow mesenchymal stem cell-derived exosomes, target PTGS2 inhibition and inhibit osteoclast differentiation, suggesting that traditional Chinese medicine is also one of the factors that alter exosome cargo [23]. To investigate the effects of exosomes from these two different sources on osteoclast differentiation, bone marrow mesenchymal stem cell exosomes from ovariectomized rats and sham rats were co-cultured with bone marrow-derived macrophages. The results showed that exosomes derived from bone marrow mesenchymal stem cells of ovariectomised rats could significantly promote osteoclast differentiation compared to sham rats, and the expression levels of miR-27a-3p and miR-196b-5p in exosomes derived from bone marrow mesenchymal stem cells of ovariectomized rats were low compared with sham rats, and it was confirmed that miR-27a-3p and miR-196b-5p negatively regulated osteoclast differentiation [24]. Additionally, a zinc-enriched microenvironment was shown to directly inhibit osteoclast differentiation and alter the exosomal cargo of senescent BMSCs, thereby modulating communication with bone marrow macrophages [25]. Innovative strategies continue to expand the therapeutic potential of exosomes. A composite hydrogel incorporating exosomes and miRNA-26a (exosome@miRNA-26a) effectively suppressed osteoclast differentiation and enhanced bone regeneration [26]. The therapeutic efficacy of MSC-derived exosomes, particularly in modulating bone-related pathways, is further evidenced by a recent meta-analysis. Kong et al. demonstrated that intra-articular injection of stem cell-derived exosomes significantly improved joint histology and reduced OARSI scores in OA animal models. These findings reinforce the broader regenerative potential of exosomes, which likely includes the modulation of osteoclast function as part of their mechanism [27].

### Adipose mesenchymal stem cells

Adipose mesenchymal stem cells, which exist in large numbers in adipose tissue, have been widely used in tissue engineering research [28]. Compared with other mesenchymal stem cells, adipose mesenchymal stem cells have many advantages, such as abundant quantity, stable function, low immune rejection and can be obtained by cell culture [29]. In 2018, Ren L et al*.* found that adipose mesenchymal stem cell-derived exosomes inhibited osteoblast-mediated osteoclast production by regulating RANKL expression and the RANKL/OPG ratio [30]. Diabetes can cause many complications, including cardiovascular disease, kidney disease, retinopathy and osteoporosis, the incidence of which is 60% higher in people with diabetes than in healthy people [31, 32]. Inflammation is a major cause of diabetic osteoporosis, and ADSC-derived exosomes have shown promise in mitigating this pathology. Zhang et al. reported that exosomes obtained from ADSCs overexpressing miRNA-146a (miR-146a-Exo) markedly inhibited osteoclastic bone resorption in vitro and attenuated hyperglycemia-induced osteoclast inflammation in vivo [33]. In a subsequent study, the same group revealed that ADSC-derived exosomes alleviate diabetic osteoporosis by suppressing NLRP3 inflammasome activation within osteoclasts [34]. Further expanding on the therapeutic potential of engineered exosomes, Hu et al. found that exosomes derived from miR-21-overexpressing ADSCs—a miRNA known to promote osteogenesis—exerted significantly stronger inhibition on osteoclast formation compared to exosomes from unmodified ADSCs [35]. Complementing these findings, Ikezaki et al. provided in vivo evidence supporting the bone-protective effects of human ADSC-derived exosomes (hADSC-exosomes). In a rabbit model of osteonecrosis of the femoral head (ONFH), local administration of hADSC-exosomes impeded disease progression, reduced osteonecrosis severity, diminished empty lacunae counts, and mitigated articular cartilage injury. Although the treatment did not significantly enhance angiogenesis or new bone formation, the study underscores the cytoprotective role of hADSC-exosomes against ischemic osteonecrosis and highlights their potential as therapeutic agents for preventing bone deterioration [36].

### Dental tissue derived stem cells

Compared with bone marrow mesenchymal stem cells, dental pulp stem cells have similar surface markers but stronger clonogenic ability [37]. At the same time, the extraordinary multi-lineage differentiation potential of dental pulp stem cells, the regulation of peridermal cells through paracrine, the ability to regulate the immune system of the microenvironment and the wide range of sources have promoted their clinical application [38]. TRPV4 is a calcium-associated transmembrane channel protein that can be activated by a variety of factors, including mechanical stress, osmotic pressure, and bioactive substances, and it has been shown that calcium influx induced by TRPV4 activation can promote osteoclast differentiation. Fu Y et al*.* found that local injection of dental pulp stem cell-derived exosomes partially reversed osteoclast activation in a mouse in vivo model. Furthermore, the anti-osteoarthritis therapeutic effect of dental pulp stem cell-derived exosomes may be achieved by inhibiting TRPV4-induced osteoclast differentiation [39]. Periodontal ligament stem cells are mechanical stress-sensitive cells, and periodontal ligament stem cells play an important role in alveolar bone reconstruction during orthodontics. Huang et al. revealed that mechanical stress can promote exosome production in periodontal ligament stem cells by upregulating RAB27B. In addition, the expression of ANX3 in exosomes derived from periodontal ligament stem cells was upregulated after mechanical stress stimulation, which promoted the phosphorylation of ERK, which in turn promoted osteoclast differentiation, and meanwhile, the upregulation of ANX3 expression also increased the uptake of exosomes by macrophages [40]. Zheng X et al*.* further reported that mechanical stress significantly alters the miRNA profile of PDLSC-derived exosomes, with bioinformatic analyses indicating these miRNAs are closely associated with osteoblast and osteoclast activity [41]. In addition, exosomes derived from periodontal ligament stem cells undergoing osteogenic differentiation can promote osteoclast production by delivering circ_0000722 to upregulate TRAF6 expression and activate NF-κB and AKT signaling pathways [42]. Periodontitis is a long-term inflammatory disease that causes bone loss in the alveolar bone. Lu J et al*.* observed that exosomes from PDLSCs cultured under normal glucose conditions inhibited osteoclast formation, whereas those from high-glucose conditions lost this inhibitory capacity—implying that hyperglycemia exacerbates periodontal disease. Mechanistically, normal glucose PDLSC-exosomes delivered miR-31-5p, which targets eNOS signaling, to suppress osteoclast formation. In a periodontitis mouse model, administration of these exosomes reduced osteoclast numbers and mitigated alveolar bone destruction [43]. Beyond PDLSCs, gingival tissue-derived mesenchymal stem cells (GMSCs) also exhibit significant immunomodulatory and osteoprotective functions.CD73 is the signature signal of mesenchymal stem cells that converts ATP to adenosine through an enzymatic reaction to mediate immunosuppression. Nakao Y et al*.* found that TNF-α pretreatment of gingival tissue-derived mesenchymal stem cells could promote the secretion of exosomes and upregulate the expression of CD73 in exosomes by mesenchymal stem cells in gingival tissue, and the upregulated CD73 could induce M2-type polarization of macrophages to alleviate periodontal tissue inflammation and bone loss. Furthermore, TNF-α stimulation elevated exosomal levels of miR-1260b, which inhibited RANKL expression via suppression of Wnt5a and JNK pathways, thereby attenuating osteoclast differentiation [44].

### Other tissue-derived stem cells

Polylactic-co-glycolic acid (PLGA) is a common drug carrier that is widely used in nanoparticle-based research due to its excellent biocompatibility, ease of production, and excellent sustained release properties. Xie J et al*.* used PLGA to encapsulate exosomes derived from human umbilical cord mesenchymal stem cells (PLGA-Exos), and found that PLGA-Exos could promote osteoblast differentiation and inhibit osteoclast differentiation in vitro experiments [45]. In addition, Guo et al. found that the expression of miR-206 was downregulated in exosomes derived from oral and facial mesenchymal stem cells knocked down by GATA4, and the downregulated miR-206 promoted osteoclast differentiation by targeting NFATc1 [46]. Beyond their regenerative effects, the practical translation of exosome therapies requires advancements in storage and handling. Lo et al. addressed this challenge by demonstrating that lyophilized (freeze-dried) exosomes derived from human umbilical cord stem cells (hUSC-EX) retained their bioactivity after hydration. In a model of chronic anterior cruciate ligament (ACL) cell injury, these readily storable exosomes significantly enhanced cell viability, proliferation, migration, and crucially, upregulated the expression of genes critical for ligament repair (Collagen I/III, TGFβ, VEGF, and tenogenesis markers). This study not only confirms the efficacy of hUSC-EX in promoting soft tissue healing but also provides a practical lyophilization strategy that facilitates the clinical storage and off-the-shelf application of exosome-based therapeutics [47]. The therapeutic potential of exosomes extends beyond osteolytic diseases. As exemplified by their novel application in modulating immune responses, mesenchymal stem cell-derived extracellular vesicles (EVs) are being investigated as an alternative to cell therapy for managing hyperinflammatory conditions like COVID-19-associated ARDS. Their proven immunomodulatory and anti-fibrotic properties position them as a versatile platform for regenerative medicine. This cross-disciplinary application underscores the broader mechanistic potential of EVs in regulating pathological immune environments—a principle that could be harnessed in the context of osteoimmunology and inflammatory bone loss [48].

## Effects of exosomes derived from osteoblasts, vascular cells and hematopoietic cells on osteoclasts

### Osteoblasts

In the skeletal system, osteoblasts and osteoclasts interact to regulate bone homeostasis. Osteoblasts can secrete cytokines such as M-CSF, RANKL and OPG, which influence osteoclast differentiation and function [53]. Emerging evidence highlights exosomes as another crucial mechanism of intercellular communication through which osteoblasts modulate osteoclast activity. Studies have revealed that osteoblast-derived exosomes can exert both inhibitory and promotive effects on osteoclastogenesis, depending on their molecular cargo. In 2021, WangQ et al*.* found that osteoblast-derived exosomes inhibited osteoclast differentiation by regulating the miR-503-3p/Hpse signaling axis [50]. WangW et al*.* found that Circ_0008542 in osteoblast exosomes can promote osteoclast differentiation and bone resorption by regulating miR-185-5p/RANK axis [51]. Beyond local bone microenvironment cues, systemic factors also influence this communication. HuCH et al*.* found that sympathetic nerve stress can trigger the transcription of miR-21 in osteoblasts through β1/2-adrenergic receptor (β1/2-AR), and miR-21 reaches progenitor osteoclasts via exosome to promote osteoclastogenesis [52]. The regulatory role of exosomal non-coding RNAs was further expanded by Zhang et al., who identified high abundance of LncRNA-MALAT1 in osteoblast-derived exosomes. This lncRNA was found to enhance osteoclast differentiation by targeting the miR-124/NFATc1 signaling axis [53]. Additionally, epitranscriptomic modifications contribute to this regulation. Yang et al. discovered that osteoblast-derived exosomes transfer METTL14, an RNA methyltransferase, which increases m6A methylation of NFATc1 mRNA—a modification that ultimately inhibits osteoclast differentiation [54]. Most recently, HakkiSS et al*.* found that osteoblast-derived exosomes promote bone formation by reducing the number of bone marrow macrophages and osteoclasts [55].

### Vascular cell

Vascular cells play an integral role in bone repair and homeostasis, not only through angiogenesis but also via paracrine signaling mediated by exosomes. CuiY et al. found that exosomes derived from endothelial progenitor cells had a higher level of MALAT1 expression than those in endothelial progenitor cells, suggesting that MALAT1 in endothelial progenitor cells may play a role by migrating into exosomes. Exosomes derived from endothelial progenitor cells were found to promote the recruitment and differentiation of osteoclast progenitors by delivering MALAT1 targeting miR-214 [56]. Pericytes, derived from the inner wall of blood vessels, influence organ development and tissue homeostasis by producing paracrine signaling molecules [57]. In the skeletal system, vascular factors secreted by pericytes and angiogenic factors secreted by bone cells co-regulate angiogenesis and bone formation. CaiM et al. demonstrated that conditional deletion of pericytes in vivo would promote bone resorption leading to bone loss. Mechanically, pericellular-derived exosomes inhibit osteoclast differentiation and bone resorption by transducing tumor necrosis factor receptor-associated factor 3 (Traf3) to inhibit NF-κB signaling [58]. Targeted therapy is one of the important ways to reduce adverse drug reactions. SongH et al. provided evidence in a mouse model that vascular endothelial cell-derived exosomes have good bone targeting and biological adaptability, and found that vascular endothelial cell-derived exosomes inhibit osteoclast differentiation and function by delivering miR-155 [59].

### Hematopoietic cells

A well-established bidirectional relationship exists between bone and hematopoiesis: bone cells regulate haematopoiesis and haematopoietic cells regulate bone remodeling [60]. Patients with chronic anaemia often experience bone loss, which has been replicated in a mouse model of anaemia. Research by Sadvakassova et al. found that exosomes released from haematopoietic cells stimulated osteoclast differentiation by delivering Peroxiredoxin-2(PRDX2) in an anaemic state [61]. Mechanical loading represents another key regulator of bone homeostasis. Emerging evidence indicates that low-intensity exercise maintains or increases bone mass, whereas lack of mechanical loading or high-intensity exercise decreases bone mass, possibly in part through the release of extracellular vesicles by mechanosensitive bone cells [62]. However, the effects of different intensities of exercise on the biological function of these vesicles have not been elucidated. Bratengeier et al. systematically addressed this question by applying precisely controlled fluid shear stress to extracellular vesicles derived from mouse hematopoietic progenitor cells. Their findings revealed a dichotomous response: vesicles subjected to low-intensity shear stress (0.7 ± 0.3 Pa, 5 Hz) for 2 min inhibited osteoclastogenesis, while those treated with high-intensity stress (2.9 ± 0.2 Pa, 1 Hz) for the same duration promoted osteoclast formation. This pioneering work demonstrates that mechanical forces can directly modify the biological activity of hematopoietic cell-derived vesicles, providing a potential mechanistic link between physical activity levels and bone remodeling [62].

## Effects of immune cell-derived exosomes on osteoclasts

Previous studies have confirmed that a large number of innate and adaptive immune cells are involved in the pathogenesis of bone diseases, where immune cells can regulate osteoclastogenesis by secreting pro- and anti-inflammatory factors and affect bone homeostasis [63]. The immune response changes depending on the maturity of the dendritic cells. Immature dendritic cells terminate the inflammatory response by inducing T cell anergy and Treg cells (regulatory T cells). However, mature dendritic cells destroy invading bacteria by activating the Th17 (T helper cell 17) response. In terms of bone physiology and pathology, Th17 cells promote bone degradation, while Treg cells inhibit the inflammatory response associated with bone loss. ElashiryM et al. found that dendritic cells loaded with anti-inflammatory factors (TGFB1 and IL-10) can inhibit dendritic cell maturation and Th17 cell activation, recruit Treg cells and inhibit osteoclast-mediated bone resorption [64]. Environmental exposures such as smoking can skew immune signaling toward osteoclast activation. DonatePB et al. revealed that smoking can induce the specific expression of miRNA-132 in Th17 cells, and miRNA-132 is packaged into EVs as a pro-inflammatory factor that promotes osteoclast formation by inhibiting the expression of COX2 [65]. Beyond adaptive immune cells, innate immune populations also modulate bone remodeling through exosomal communication. M2 macrophages, known for their anti-inflammatory and tissue-reparative functions, release exosomes that exert inhibitory effects on osteoclastogenesis. ChenX et al. found that M2 macrophage-derived exosomes inhibited osteoclast formation by delivering IL-10 to bone marrow-derived macrophages [66]. Similarly, ZhouY et al. reported that M2-type macrophage-derived exosomes inhibit the activation of TNF-α signaling by downregulating the expression of CSF2, thereby inhibiting RANKL-induced osteoclast differentiation [67].

## Effects of tumour cell-derived exosomes on osteoclasts

**Fig. 2:**
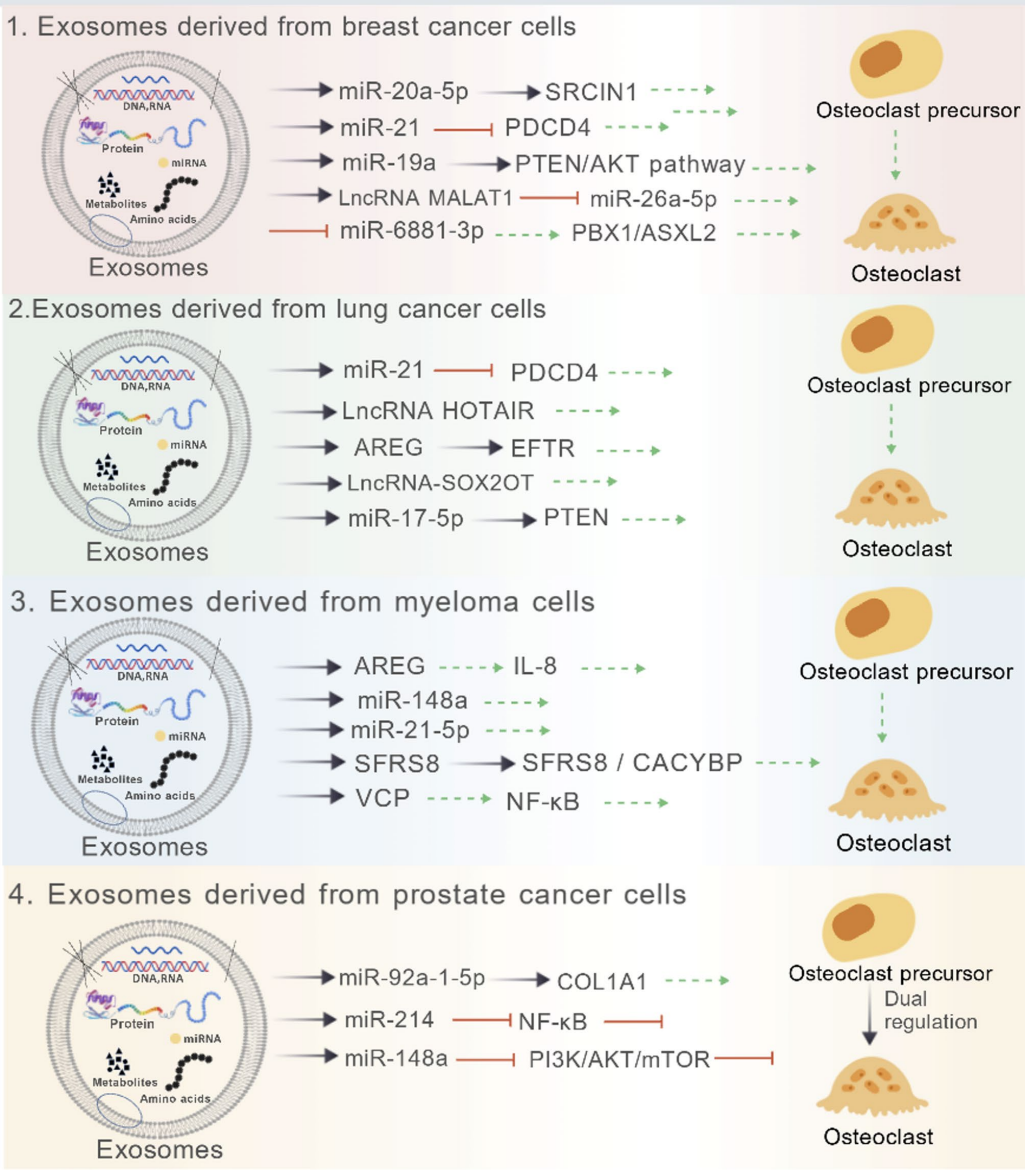
Effects of tumor cell-derived exosomes on osteoclasts. (Created with BioGDP.com [49]). All molecules shown at the origin of the arrows (e.g., miR-20a-5p, LncRNA MALAT1) represent the specific cargo encapsulated within the exosomes derived from different tumor cell types. The arrowheads indicate the downstream molecules or pathways that are targeted and regulated by this exosomal cargo in the recipient osteoclast precursors. (Black solid arrows: Targeted delivery relationships; Red T-bar arrows: Inhibition of osteoclast differentiation; Green dashed arrows: Promotion of osteoclast differentiation. Notably, exosomes derived from prostate cancer cells exert dual regulation on osteoclasts, involving both promoting and inhibiting effects.)

Tumor-derived exosomes are increasingly recognized as critical mediators in the bone metastatic niche, capable of reprogramming osteoclast precursors and driving osteolytic destruction through the transfer of oncogenic miRNAs, proteins, and lncRNAs. The following sections detail how exosomes from specific cancers—such as breast, lung, and prostate—modulate osteoclastogenesis, as summarized in Fig. 2.

### Breast cancer cells

Breast cancer is the most common malignancy in women and exhibits a high propensity for metastasis to bone, leading to severe osteolytic lesions and significant morbidity. The role of tumor-derived exosomes in preparing the pre-metastatic niche and promoting osteoclast activation is now recognized as a critical step in this process [68, 69]. In the context of breast cancer bone metastasis, exosomal cargo has been identified as a key mediator. For instance,in 2019, GuoL et al*.* found a high expression level of miR-20a-5p in human breast cancer cells and their exosomes. Clinical data showed that the expression level of miR-20a-5p was higher in patients with bone metastasis than in patients without bone metastasis. Breast cancer cell-derived exosomes were found to deliver miR-20a-5p targeting SRCIN1 to promote osteoclast proliferation and differentiation and to promote breast cancer bone metastasis [70]. Similarly,YuanX et al*.* reported that breast cancer-derived exosomes promoted osteoclast differentiation by delivering miRNA-21 targeted inhibition of PDCD4 [71]. Among breast cancer subtypes, patients with estrogen receptor^+^ (ER^+^) breast cancer had the highest incidence of bone metastases. Integrin-binding sialoprotein (IBSP) is the major binding protein of the bone matrix and is mainly secreted by bone cells [72]. WuK et al*.* found that the expression of miRNA-19a and IBSP was significantly upregulated in exosomes derived from ER^+^ breast cancer cells. Further studies have shown that miRNA-19a promotes osteoclast formation by regulating the PTEN/AKT pathway, while IBSP can recruit osteoclast progenitor cells to aggregate [72]. Recent investigations have further expanded our understanding of exosome-mediated mechanisms in different molecular contexts. In 2023, XuM et al*.* found that JAG1 can promote the secretion of exosomes by triple-negative breast cancer cells and upregulate the expression level of LncRNA MALAT1 in the exosomes, and LncRNA MALAT1 promotes macrophage adhesion, migration and osteoclast differentiation by targeting the inhibition of miR-26a-5p [73]. Additionally, LSD1, a tumor suppressor frequently downregulated in breast cancer, modulates exosomal content [74]. LiuZ et al*.* found that the expression of miR-6881-3p is downregulated in exosomes from breast cancer cells knocked down by LSD1, thereby upregulating the expression of its target genes PBX1 and ASXL2 to promote osteoclast differentiation [75]. In addition, RAS activation is critical for breast cancer invasion and metastasis [76]. The RAS signaling pathway, crucial for breast cancer invasion and metastasis, also influences exosome composition. KimO et al*.* demonstrated that RAS-activated exosomes derived from breast cancer cells can promote osteoclast formation and thus promote bone metastasis of breast cancer by transferring miR-494-3p [77]. Collectively, these findings underscore the multifaceted role of breast cancer-derived exosomes in bone metastasis through diverse molecular mechanisms, highlighting their potential as therapeutic targets for preventing and treating bone metastatic disease.

### Lung cancer cells

30–40% of patients with advanced lung cancer have bone metastases, and complications such as pathological fractures can occur [78]. During bone metastasis of cancer, once the bone matrix is dissolved by osteoclasts, the release of growth factors stored in the bone matrix into the environment promotes the proliferation of cancer cells [79]. Exosomes secreted by lung cancer cells play a crucial role in this process by transferring osteoclast-activating molecules. In vitro experiments, XuZ et al*.* found that lung cancer cells can promote osteoclast formation, and lung cancer cells and their secreted exosome miR-21 expression levels are higher than normal lung cells. Mechanistic studies have shown that exosomes derived from lung cancer cells promote osteoclast generation by transmitting miR-21 targeting PDCD4 signals [80]. This study showed that cancer cells promote osteoclastogenesis through the transfer of exosomes, and the growth factors released after bone matrix erosion will promote tumor cell proliferation, creating a vicious cycle that promotes cancer cell proliferation and invasion. Further supporting this mechanism, ZhangC et al*.* reported that exosomes derived from lung cancer cells promote osteoclast differentiation by delivering LncRNA HOTAIR targeting TGF-β/PTHrP/RANKL signaling pathway [81]. The epidermal growth factor receptor (EGFR) signaling pathway is active in non-small cell lung cancer (NSCLC) [82]. TavernaS et al. found that NSCLC-derived exosomes activate the EGFR signaling pathway by transducing AREG, thereby inducing RANKL expression and promoting osteoclastogenesis [68]. Furthermore, NiJ et al*.* found a high abundance of LncRNA-SOX2OT in NSCLC-derived exosomes, and LncRNA-SOX2OT promoted osteoclast differentiation by targeting the miRNA-1945p/RAC1 signaling axis and the TGF-β/pTHrP/RANKL signaling pathway [83]. WangM et al*.* further contributed to this understanding by showing that exosomes derived from NSCLC cells promote osteoclastogenesis by delivering miR-17-5p targeting PTEN [84].

### Myeloma cells

In multiple myeloma, bone destruction is driven by a complex interplay of cytokines and exosome-mediated signaling. Interleukin-8 (IL-8) contributes significantly to osteolysis in this setting [85]. Exosomes secreted by multiple myeloma cells outside the body of EGFR ligand double regulatory proteins (AREG) abundance is high. Experimental studies have found that AREG in multiple myeloma-derived exosomes can promote the secretion of IL-8 by human mesenchymal stromal cells and induce the formation of osteoclasts. In addition, multiple myeloma-derived exosomes can increase the rate of RANKL/OPG secretion by human mesenchymal stromal cells, but AREG is not regulated [86]. The EVs of the tumor can be distributed to the distal tissues through the blood, and the mice can induce osteolysis after intravenous injection of EVs derived from mouse myeloma cells 5TGM1 [87]. In vitro experiments, EVs derived from 5TGM1 cells induced the differentiation of mouse macrophages Raw246.7 into osteoclasts and increased their bone resorption capacity. In a tumour-bearing mouse model of 5TGM1 cells, the exosome secretion inhibitor GW4869 not only reduced osteolysis, but also sensitized myeloma cells to bortezomib when combined with the anti-cancer drug bortezomib, thereby enhancing the anti-tumor efficacy of bortezomib. This study showed that myeloma cells promoted osteolysis by secreting exosomes, and that exosomes secreted by myeloma cells were associated with drug resistance in myeloma [88]. In addition, RaimondiL et al*.* found that multiple myeloma cell-derived exosomes can promote osteoclast differentiation by inducing the expression of osteoclast differentiation genes, including cathepsin K (CTSK), matrix metalloproteinase 9 (MMP9), and tartrate-resistant acid phosphatase (TRAP) [89]. The same group also identified miR-148a and miR-21-5p as pro-osteoclastogenic miRNAs delivered by myeloma exosomes [90]. ZhangY et al. further found that multiple myeloma cell-derived exosomes promote osteoclast differentiation by delivering splicing factor arginine/serine-rich 8 (SFRS8) and targeting the SFRS8/calcyclin-binding protein (CACYBP)/β-catenin axis [91]. Complementing these findings, WeiR et al. demonstrated that multiple myeloma cell-derived exosomes promote osteoclast differentiation by delivering valosin-containing protein (VCP) and activating the NF-κB signaling pathway [92].

### Prostate cancer cells

Because the early symptoms of prostate cancer are not obvious, it is difficult for patients to receive effective treatment in the early stages, so prostate cancer often progresses to an advanced stage. Bone metastases are common in advanced prostate cancer, and 70–80% of men diagnosed with castration-resistant prostate cancer have bone metastases on imaging [93]. YuL et al*.* confirmed in vivo experiments that exosomes derived from prostate cancer cells have a stronger bone targeting effect compared to normal prostate cells. In addition, prostate cancer cell-derived exosomes can promote osteoclast differentiation without the assistance of RANKL, suggesting that prostate cancer cell-derived exosomes can activate non-RANKL-dependent pathways that promote osteoclast differentiation. Mechanistically, miRNA-92a-1-5p in prostate cancer-derived exosomes targets COL1A1 to promote osteoclast differentiation and inhibit osteoblast differentiation, thereby promoting bone metastasis of prostate cancer [94]. Paradoxically, several studies have reported inhibitory effects of prostate cancer-derived exosomes on osteoclast formation. In 2016, KarlssonT et al*.* observed that exosomes derived from TRAMP-C1 mouse prostate cancer cells significantly inhibited osteoclast differentiation [95]. Subsequent investigations revealed additional mechanisms underlying this inhibition: DuanY et al*.* found that exosomes derived from PC-3 prostate cancer cells inhibited osteoclast differentiation by downregulating miR-214 to inhibit the NF-κB signaling pathway [96]. Similarly, TianG et al. demonstrated that PC-3 cells-derived exosomes inhibited osteoclast differentiation by downregulating miR-148a and blocking the PI3K/AKT/mTOR signaling pathway [83]. These contradictory findings may reflect differences in cancer cell subtypes, experimental conditions, or temporal context of exosome secretion. Alternatively, prostate cancer cells might employ exosome-independent mechanisms to promote osteoclast differentiation in certain circumstances. Further research is needed to elucidate the context-dependent roles of prostate cancer exosomes in bone remodeling.

### Other cancer cells

Beyond the commonly studied carcinomas, exosomes derived from other cancer types also contribute to bone metastasis by actively promoting osteoclast differentiation and activity [97]. Pituitary carcinoma, though rare, demonstrates this capacity through exosome-mediated mechanisms. In 2020, WangT et al*.* found that pituitary carcinoma-derived exosome activated PI3K-AKT, MAPK and calcium signaling pathways through the RASSF10/MDM2 pathway, thereby promoting osteoclast differentiation and causing bone invasion of pituitary cancer [98]. Similarly, ZhouY et al*.* revealed that pancreatic cancer-derived exosomes promote osteoclast differentiation by transferring miR-125-5p and inhibiting TNFRSF1B [99]. In bone-related malignancies, osteosarcoma-derived exosomes exhibit particularly potent osteoclast-stimulating effects. LinL et al*.* found that osteosarcoma cell-derived exosomes promote osteoclast differentiation and exacerbate bone loss by delivering miR-501-3p targeting PTEN [100, 101]. Hepatocellular carcinoma also employs exosomal signaling to manipulate bone homeostasis. LiCH et al*.* found that TNF-α in Huh-7-derived exosomes from liver cancer cells promoted osteoclast differentiation by upregulating the expression of the osteoclast marker NF-κB/CTSK/TRAP [101]. More recently, Li et al. identified an additional mechanism whereby liver cancer-derived exosomes deliver miR-574-3p to inhibit BMP2 signaling, further stimulating osteoclastogenesis [102]. These findings across multiple cancer types underscore the universal importance of exosome-mediated communication in bone metastasis and highlight the diverse molecular strategies tumors employ to activate osteoclasts and disrupt skeletal homeostasis.

## Effects of exosomes derived from other cells on osteoclasts

Synovial fibroblasts are a type of cell in the synovial tissue that is involved in maintaining the normal function of the joints and is also the main site of lesion in several joint diseases [103]. Synovial fibroblasts have long been considered a potential therapeutic target in rheumatoid arthritis, but there are still no approved treatments for synovial fibroblasts in rheumatoid arthritis [104]. Emerging evidence implicates exosomes derived from synovial fibroblasts in disease mechanisms. For instance, TakamuraY et al. found that after TNF-α stimulation, miR-1307-3p was upregulated in fibroblast-derived exosomes, and the mechanism was that miR-1307-3p in exosomes was transferred to monocytes to promote osteoclast differentiation and cause joint destruction by targeting NDRG2 [105]. These findings suggest that modulating miR-1307-3p cargo in synovial fibroblast-derived exosomes could offer an indirect therapeutic strategy for managing RA and similar joint diseases. Furthermore, the potential of synovial fluid-derived exosomes (SF-exo) as a rich source of biomarkers for joint diseases like osteoarthritis (OA) is increasingly recognized. A recent quantitative proteomic study by Wu et al. provided a comprehensive analysis of SF-exo from OA patients and healthy controls. They identified 25 differentially expressed proteins (DEPs), with 20 upregulated and 5 downregulated in OA. Bioinformatic analysis revealed that these DEPs were significantly enriched in immune-related processes such as complement activation (e.g., C3, C4B) and antigen binding. By integrating with a public dataset, they pinpointed C3, C4B, and APOM as core components of a key protein-interaction network. This study not only delineates the specific proteomic profile of OA SF-exo but also strongly supports the broader concept that exosomes carry molecular signatures reflective of pathological states, offering immense value for the diagnosis and therapeutic monitoring of osteolytic joint diseases [106]. Exosomes from other cellular sources also significantly influence osteoclast activity. For example, in periodontitis models, endodontic cell-derived exosomes mitigated alveolar bone resorption and suppressed osteoclast formation in vitro [107]. Conversely, keratinocyte-derived exosomes carrying miRNA-17 enhance RANKL expression in fibroblasts, thereby promoting osteoclast differentiation [108]. Age-related changes also alter exosomal cargo: compared with young people, ICAM1 and SAA1 are upregulated in serum-derived exosomes in the elderly, while PRDX2 is downregulated. Among them, ICAM1 can promote osteoclast function, SAA1 induces the production of MMP9 to promote bone resorption, and PRDX2 can resist oxidative damage at the stage of osteoclast formation. Upregulation of TGF-β inhibits osteoclast function during osteoclast bone resorption, and cytoplasmic ribosomal proteins are upregulated during RANKL-induced osteoclast differentiation. XieY et al. also found that TGF-β was downregulated and cytoplasmic ribosomal protein was upregulated in serum-derived exosomes in elderly patients with osteoporosis or osteopenia compared to healthy elderly patients, thereby promoting osteoclast differentiation and function [109]. Exosomal involvement is also evident in genetic and systemic disorders. Deletion of the NF1 gene can affect bone development, leading to bone deformities, and it has been well established that children with congenital tibial pseudoarthrosis are closely linked to the inactive state of the NF1 gene. At present, the pathogenesis of congenital tibial pseudoarthrosis is not clear. YangG et al. found that serum-derived exosomes can promote osteoclast differentiation in patients with congenital tibial pseudoarthrosis. In addition, bioinformatics analysis showed that changes in bone remodelling may be associated with altered protein expression profiles and biological processes [110]. Osteoclasts can form an actin ring that enables them to adhere tightly to the bone surface. Changes in the structure of the actin ring will encourage osteoclasts to resorb bone matrix more efficiently. Exosomes in the synovial fluid of patients with rheumatoid arthritis can induce osteoclast differentiation, and cytoskeletal rearrangement and expression of RANKL on the exosomal surface may be the reasons for promoting osteoclast differentiation [111]. Patients with chronic kidney disease are prone to renal osteodystrophy, in which inflammation is closely associated with bone loss caused by renal osteodystrophy. FuR et al*.* found that renal osteodystrophy can alter the miRNA cargo in bone marrow-derived exosomes and promote inflammation and osteoclast differentiation [112]. Notably, certain natural exosomes exhibit inhibitory effects on osteoclasts. Bovine colostrum-derived exosomes, which share miRNA homology with humans, suppress osteoclast differentiation and prevent glucocorticoid-induced osteoporosis in mice, suggesting dietary milk intake may benefit patients on glucocorticoid therapy [113]. This suggests that patients who are undergoing glucocorticoid therapy can take an appropriate amount of milk daily to prevent glucocorticoid-induced osteoporosis. Previous studies have reported that plums and prunes contain high levels of polyphenols that can improve bone mass and density and inhibit osteoclast differentiation by inhibiting RANKL signaling. ParkYS et al*.* found that plum-derived exosome-like vesicles could inhibit osteoclast differentiation [114]. Liu et al. demonstrated that exosomes derived from platelet-rich plasma (PRP-exos) could effectively promote cartilage repair. When combined with a cyclic peptide-modified β-TCP scaffold, PRP-exos implantation in a rabbit knee cartilage defect model significantly upregulated cartilage-specific markers (COL-2, SOX9, RUNX2), downregulated inflammatory cytokines (TNF-α, IL-1β, IL-6) and catabolic factor MMP-3, while increasing its inhibitor TIMP-1. miRNA sequencing and bioinformatic analysis suggested that the PI3K/AKT signaling pathway may be a key mechanism mediating these reparative effects. This study highlights PRP as a novel and clinically accessible source of exosomes for enhancing cartilage regeneration [115].

## Conclusion and prospect

### Summary and significance

This review has synthesized compelling evidence establishing exosomes as pivotal mediators of osteoclast function and bone homeostasis. We have delineated how exosomes from a diverse array of cellular sources—including stem cells, osteoblasts, vascular cells, immune cells, and tumor cells, as comprehensively summarized in Table 1—orchestrate osteoclastogenesis through the delivery of specific molecular cargo (e.g., miRNAs, lncRNAs, proteins). A key recurring theme is the dualistic nature of exosomes: while those from stem and osteoblastic lineages typically exert inhibitory effects on osteoclast formation, exosomes derived from tumor and inflamed immune cells potently promote bone resorption, highlighting their role in both pathological destruction and potential repair. The recent proteomic profiling of synovial fluid exosomes further underscores their value as reservoirs of disease-specific biomarkers [52], while meta-analyses corroborate the robust therapeutic efficacy of MSC-exosomes in pre-clinical models of joint disease [57].

**Table 1:** Summary of the effects of exosomes from different cellular origins on osteoclasts

Exosome source	Key exosomal cargo		Targeted pathway/gene		Effect on osteoclasts	
*Stem cells*						
Aged BMSCs	miR-31a-5p		RhoA		Promotes differentiation	
Engineered BMSCs	BT-Exo-siShn3		Shn3		Inhibits differentiation	
Engineered BMSCs (Scleraxis-OE)	miR-6924-5p		OCSTAMP, CXCL12		Inhibits formation	
Morinda officinalis-treated BMSCs	miR-101-3p		PTGS2		Inhibits differentiation	
ADSCs	(Carg not specified)		RANKL/OPG Ratio		Inhibits production	
ADSCs (miR-146a-OE)	miR-146a		NLRP3 Inflammasome		Inhibits differentiation & Inhibits resorption	
ADSCs (miR-21-OE)	miR-21		(Not specified)		Inhibits formation	
Dental pulp stem cells (DPSCs)	(Cargo not specified)		TRPV4		Inhibits activation & Inhibits differentiation	
Periodontal Ligament stem cells (PDLSCs, mechanical stress)	ANXA3 (Protein)		ERK		Promotes differentiation	
PDLSCs (osteogenic differentiation)	circ_0000722		TRAF6/NF-κB/AKT		Promotes production	
PDLSCs (normal glucose)	miR-31-5p		eNOS		Inhibits formation	
Gingival MSCs (TNF-α pretreated)	CD73 (Protein), miR-1260b		Wnt5a/JNK		Inhibits differentiation	
Orofacial MSCs (GATA4 knockdown)	miR-206 (Downregulated)		NFATc1		Promotes differentiation	
*Osteoblasts*						
Osteoblasts	miR-503-3p		Hpse		Inhibits differentiation	
Osteoblasts	Circ_0008542		miR-185-5p/RANK		Promotes differentiation	
Osteoblasts (sympathetic stress)	miR-21		(Not specified)		Promotes osteoclastogenesis	
Osteoblasts	LncRNA-MALAT1		miR-124/NFATc1		Promotes differentiation	
Osteoblasts	METTL14 (Protein)		NFATc1 m6A Methylation		Inhibits differentiation	
*Vascularcells*						
Endothelial progenitor cells (EPCs)	LncRNA-MALAT1		miR-214		Promotes recruitment	
Vascular pericytes	Traf3 (Protein)		NF-κB		Inhibits differentiation	
Vascular endothelial cells	miR-155		(Not specified)		Inhibits differentiation	
*Hematopoietic cells*						
Hematopoietic cells (anemia)		PRDX2 (Protein)		(Not specified)		Promotes differentiation
Hematopoietic progenitors (low-intensity stimulation)		(Cargo altered)		(Not specified)		Inhibits osteoclastogenesis
Hematopoietic progenitors (high-intensity stimulation)		(Cargo altered)		(Not specified)		Induces formation
*Immune cells*						
Dendritic cells (loaded with anti-inflammatory factors)		TGFB1, IL-10 (Protein)		(Modulates T-cell response)		Inhibits bone resorption
Th17 cells (smoke-induced)		miRNA-132		COX2		Promotes formation
M2 macrophages		IL-10 mRNA		(Not specified)		Inhibits formation
M2 macrophages		(Cargo not specified)		CSF2/TNF-α Axis		Inhibits differentiation
*Tumor cells*						
Breast cancer cells		miR-20a-5p		SRCIN1		Promotes proliferation & differentiation
Breast cancer cells		miRNA-21		PDCD4		Promotes differentiation
ER + breast cancer cells		miRNA-19a, IBSP (Protein)		PTEN/AKT		Promotes formation & Progenitor recruitment
Triple-negative breast cancer cells		LncRNA MALAT1		miR-26a-5p		Promotes differentiation
Breast cancer cells (LSD1 knockdown)		miR-6881-3p (Downregulated)		PBX1, ASXL2		Promotes differentiation
RAS-activated breast Cancer cells		miR-494-3p		(Not specified)		Promotes formation
Lung adenocarcinoma cells		miR-21		PDCD4		Promotes generation
Lung cancer cells		LncRNA HOTAIR		TGF-β/PTHrP/RANKL		Promotes differentiation
NSCLC cells		LncRNA-SOX2OT		miRNA-194-5p/RAC1, TGF-β/pTHrP/RANKL		Promotes differentiation
NSCLC cells		miR-17-5p		PTEN		Promotes osteoclastogenesis
Multiple myeloma (MM) cells		AREG (Protein)		IL-8		Induces formation
Multiple myeloma (MM) cells		(Cargo not specified)		(Increases RANKL/OPG Ratio)		Promotes generation
Myeloma cells		miR-148a, miR-21-5p		(Not specified)		Promotes differentiation & resorption
Myeloma cells		SFRS8 (Protein)		SFRS8/CACYBP/β-catenin Axis		Promotes differentiation
Myeloma cells		VCP (Protein)		NF-κB		Promotes differentiation
Prostate cancer cells		miRNA-92a-1-5p		COL1A1		Promotes differentiation
Prostate cancer cells (TRAMP-C1)		(Cargo altered)		(Not specified)		Inhibits formation & differentiation
Prostate cancer cells (PC-3)		(miR-214 Downregulated)		NF-κB		Inhibits differentiation
Prostate cancer cells (PC-3)		(miR-148a Downregulated)		PI3K/AKT/mTOR		Inhibits differentiation
Pituitary adenoma cells		(Via RASSF10/MDM2)		PI3K-AKT, MAPK, Calcium Signaling		Promotes differentiation
Pancreatic cancer cells		miR-125a-5p		TNFRSF1B		Promotes differentiation
Osteosarcoma cells		miR-501-3p		PTEN		Promotes differentiation & exacerbates bone loss
Hepatocellular carcinoma cells (Huh-7)		TNF-α (Protein)		(Upregulates NF-κB/CTSK/TRAP)		Promotes differentiation
Liver cancer cells		miR-574-3p		BMP2		Promotes differentiation
*Other cells*						
Synovial fibroblasts (TNF-α stimulated)		miR-1307-3p		NDRG2		Promotes differentiation
Keratinocytes		miRNA-17		(RANKL in Fibroblasts)		Promotes differentiation
Serum (elderly individuals)		ICAM1, SAA1 (Up); PRDX2 (Down)		(Multiple mechanisms)		Promotes function & differentiation
Serum (NF1 pseudarthrosis patients)		(Altered Protein Profile)		(Not specified)		Promotes differentiation
Synovial fluid (rheumatoid arthritis)		(Surface RANKL, etc.)		(Cytoskeletal Rearrangement)		Induces differentiation
Bone marrow (renal osteodystrophy)		(Altered miRNA Cargo)		(Pro-inflammatory)		Promotes differentiation
Bovine colostrum		(Cargo not specified)		(Not specified)		Inhibits differentiation
Plum		(Polyphenols, etc.)		(RANKL Signaling Inhibition)		Inhibits differentiation
Platelet-Rich Plasma (PRP)		(Various cargo, e.g., miRNAs)		PI3K/AKT (Putative)		(Promotes cartilage repair, indirect effect)

### Current challenges and limitations

Despite the promising outlook, several formidable challenges must be acknowledged before clinical translation can be realized. A primary limitation lies in the heterogeneity of exosome isolation and characterization methods across studies, leading to inconsistencies in particle concentration, purity, and functionality, which complicates the comparison of results and replication of findings. Furthermore, while studies like that of Ikezaki et al. demonstrate efficacy in preventing osteonecrosis, critical gaps remain in our understanding of the in vivo pharmacokinetics, biodistribution, and long-term safety profiles of administered exosomes [36]. Most current strategies, including the lyophilization approach presented by Lo et al., lack specific targeting mechanisms to direct exosomes to bone tissue, resulting in potential off-target effects and reduced therapeutic efficacy at the desired site. Moreover, the scalable manufacturing of clinical-grade exosomes under Good Manufacturing Practice (GMP) standards remains a significant economic and technical hurdle [47].

### Future perspectives and clinical translation

Future research must pivot from exploratory discovery to translational development. Key priorities should include: (1) Standardization and Targeted Engineering: There is an urgent need for uniform protocols in exosome isolation, quantification, and functional assessment. Engineering exosomes with bone-targeting motifs (e.g., BT-Exo [19]) or loading them with specific anti-osteoclastogenic molecules (e.g., siShn3 [19], miR-146a [34]) represents a promising approach to enhance specificity and potency. Furthermore, the regenerative application of exosomes is evolving beyond purified vesicle preparations. Innovative cell-free strategies, such as the stem cell-derived extract (CCM) formulated by Gupta et al., exemplify this advancement. This complex formulation, derived from human progenitor endothelial stem cells (hPESCs), contains a cocktail of bioactive factors—including growth factors (VEGF, PDGF-AA, TGF-β1), anti-inflammatory cytokines (IL-1RA), and exosomes (CD81 + , CD9 +) [116]. (2)Deepening Mechanistic Understanding: Future studies should leverage multi-omics integration and advanced genetic tools, such as CRISPR-Cas9, to establish causal links between exosomal cargo and downstream pathways regulating osteoclast behavior [117]. (3) Innovative Delivery Strategies: Developing advanced delivery systems—such as PRP-exos combined with β-TCP scaffolds or stabilized lyophilized formulations—will be essential to improve stability, control release, and facilitate clinical adoption. The creation of “off-the-shelf” allogeneic exosome products could significantly broaden accessibility [118]. (4) Rigorous Pre-clinical and Clinical Validation: Well-designed large-animal studies and subsequent early-phase clinical trials are imperative to validate safety and efficacy in humans. Efforts should focus on defining optimal dosing regimens, administration routes, and patient stratification biomarkers [119]. In summary, exosomes offer considerable therapeutic potential for the treatment of osteolytic diseases through precise regulation of osteoclast function. Strategically modifying exosomal cargo or employing exogenous stimuli may further enhance their efficacy. With continued research and methodological refinement, exosome-based therapies are poised to become valuable biological tools in clinical orthopedics, ultimately offering new hope for patients with bone destructive disorders.

## Data Availability

No datasets were generated or analysed during the current study.
